# The History of the Intron—Balancing Gains and Losses

**DOI:** 10.1371/journal.pbio.0020447

**Published:** 2004-11-30

In the 25 years since they were first discovered, introns have puzzled molecular biologists because of their uncertain function and mysterious origin. Introns are non-coding DNA sequences that reside inside a gene, splitting it into discrete units called exons. The resulting disruption of coding sequence continuity would wreak havoc in protein assembly if eukaryotic cells did not dispose of introns in messenger RNAs—the intermediates in the decoding of gene sequences to produce protein chains—in a now well-described process known as splicing.

At first glance, introns may seem like pesky parasites for which eukaryotes have cleverly evolved bypass mechanisms. But introns may also benefit their hosts. Evolutionary advantages of introns include the possibility to create new genes by cutting and pasting exons from existing genes or to diversify the protein output of a single gene by splicing the exons together in different ways. Thus, balancing intron gains and losses clearly has important evolutionary implications for a host.

Yet different organisms strike that balance differently. The budding yeast Saccharomyces cerevisiae averages less than one intron per gene, whereas mammalian genes routinely have 10 or more. Whether these differences reflect different propensities for gaining or losing introns is the subject of ongoing debates.

Organisms with low intron density display a bias for insertions at the beginning (5′ end) rather than the end (3′ end) of genes. A popular hypothesis is that in these organisms, genes lose their introns through a process that rewrites genomic DNA using as template the messenger RNAs purged of intron sequences. This process might preferentially remove 3′ introns because it relies on an enzyme called reverse transcriptase that can be primed to read RNAs starting at their 3′ end. The hypothesis has gained experimental support in yeast. It also presents the advantage of potentially explaining intron paucity and 5′ position bias in one stroke. In a new study, Cydney Nielsen and her colleagues present evidence that challenges this model.

They address intron dynamics with a genome-wide survey of intron distribution among four Ascomycete fungi with recently completed genome sequences. The four fungi (Neurospora crassa, Magnoporthe grisea, Fusarium gramineum, and Aspergillus nidulans) form an evolutionary tree with branching points estimated at 200, 230, and 330 million years ago. While they diverged from yeast some 500 million years ago, they share with yeast a low intron density (one to two introns per gene) and a 5′ position bias. The authors' approach is to tally intron gains and losses during the evolution of these four species and then plot their positions along the genes' length.

They identify 3,450 gene regions that are clearly conserved in all four species and harbor an intron in at least one of them. To distinguish intron gains from losses, they rely on a simple parsimony principle, which they refine with additional probability analyses. In brief, an intron present in only one species counts as a gain; an intron absent from one species but present in its closest relative and in a cousin counts as a loss.

Nielsen and colleagues record between 150 and 350 intron losses in each lineage. Surprisingly, losses do not occur preferentially at the genes' 3′ end. The authors conclude that while a 3′ reverse transcriptase-based mechanism might be a factor, it cannot be the sole reason for the introns' 5′ bias. The other surprising result is that intron gains occur at almost the same rate as losses in all lineages. Intron gains therefore play an important role in the evolution of even intron-poor genomes. Clearly, intron distribution in fungi owes to forces more complex than simple 3′ intron elimination, forces that the authors propose may also shape evolution of other eukaryotic genomes.

